# ﻿A new species of the genus *Scincella* Mittleman, 1950 (Squamata, Scincidae) from Guizhou Province, southwest China

**DOI:** 10.3897/zookeys.1258.161382

**Published:** 2025-11-03

**Authors:** Yuhao Xu, Zhonghao Gong, Tan Van Nguyen, Jundong Deng, Andrey M. Bragin, Shiyang Weng, Tierui Zhang, Nikolay A. Poyarkov, Lifang Peng

**Affiliations:** 1 State Key Laboratory of Plateau Ecology and Agriculture, Qinghai University, Xining 810016, Qinghai, China; 2 The School of Medicine & Pharmacy, Duy Tan University, Da Nang, 550000, Vietnam; 3 Center for Entomology & Parasitology Research, Duy Tan University, Da Nang, 550000, Vietnam; 4 Joint Vietnam - Russia Tropical Science and Technology Research Centre, 63 Nguyen Van Huyen Road, Nghia Do, Hanoi, Vietnam; 5 Institute of Plateau Biology of Xizang Autonomous Region, Lhasa 850008, China; 6 Anhui Province Key Laboratory of the Conservation and Exploitation of Biological Resource, College of Life Sciences, Anhui Normal University, Wuhu 241000, Anhui, China; 7 Department of Vertebrate Zoology, Lomonosov Moscow State University, Leninskiye Gory, GSP–1, Moscow 119991, Russia

**Keywords:** Molecular phylogeny, morphology, *Scincella tenuistriata* sp. nov., taxonomy

## Abstract

A new species of the genus *Scincella* Mittleman is described from Qixingguan District, Bijie City, Guizhou Province, China, based on morphological and molecular evidence. The new species, *Scincella tenuistriata***sp. nov.**, is diagnosed by the medium body size; tympanum diameter significantly larger than the palpebral disc; midbody scale rows 24; total ventral + gular scale rows numbering 61–66; toes nearly or just touching fingers when limbs are adpressed; 9 or 10 enlarged lamellae beneath finger IV, and 11 or 12 beneath toe IV; and the dark dorsolateral stripes narrow and wavy, covering 0.5–1 scale rows on the trunk, with four scale rows in between on the dorsum. Phylogenetic analyses based on the mitochondrial *12S*, *16S*, and *CO1* gene fragments indicate that the new species is most closely related to *S. alia* Bragin, Zenin, Nguyen & Poyarkov, but differs by an uncorrected *p*-distance of 8.6–8.8% in the *CO1* gene. The discovery of the new species raises the number of currently recognized *Scincella* species to 51, underscoring the underestimated diversity of the genus.

## ﻿Introduction

The genus *Scincella* Mittleman, comprises small, terrestrial skinks distributed widely across regions from North America to South, East, and Southeast Asia (Ouboter 1986; Zhao et al. 1999; Uetz et al. 2025). Currently, 50 species of *Scincella* have been documented, including 16 species recorded from China, namely *Scincella barbouri* (Stejneger), *S. chengduensis* Jia, Gao, Wu, Wang, Liu, Liu, Jiang, Jiang, Ren & Li, *S. doriae* (Boulenger), *S. fansipanensis* Okabe, Motokawa, Koizumi, Nguyen, Nguyen & Bui; *S. formosensis* (Van Denburgh), *S. huanrenensis* Zhao & Huang, *S. liangshanensis* Jia, Gao, Wu, Ren, Jiang & Wu, *S. modesta* (Günther), *S. monticola* (Schmidt), *S. potanini* (Günther), *S. przewalskii* (Bedriaga), *S. qianica* Xu, Weng, Poyarkov, Zhang, Deng & Peng, *S. reevesii* (Gray), *S. schmidti* (Barbour), *S. tsinlingensis* (Hu & Zhao), and *S. wangyuezhaoi* Jia, Gao, Huang, Ren, Jiang & Li (Uetz et al. 2025; Xu et al. 2025a, b). This positions China as one of the major centers of diversity for the genus *Scincella* (Jia et al. 2024, 2025; Bragin et al. 2025a, b; Nguyen et al. 2025; Uetz et al. 2025). Morphologically, members of *Scincella* can be distinguished from other skinks by a unique combination of characters: a small and slender body, short limbs, absence of supranasals, lower eyelid with transparent or opaque disc, limbs pentadactyl, one row of lamellae under the basal digits, and the lower secondary temporal overlapping the upper one (Greer 1974; Greer and Shea 2003; Nguyen et al. 2010a). Species of this genus are highly adaptable and inhabit a wide range of environments, including forests, stream margins, hillsides, and arid valleys (Jia et al. 2023, 2024; Xu et al. 2025a, b).

The recent application of integrative taxonomy, which combines molecular and morphological data, has significantly advanced our understanding of the genus *Scincella*, resulting in the description of more than twelve new species over the past five years (e.g., Nguyen et al. 2020; Koizumi et al. 2022; Jia et al. 2023, 2024; Okabe et al. 2024; Pham et al. 2024, 2025; Bragin et al. 2025a, b; Jia et al. 2025; Nguyen et al. 2025; Xu et al. 2025b). Despite these advances, several taxonomic challenges remain unresolved. The inter – and intraspecific relationships within widely distributed species such as *S. doriae*, *S. melanosticta* (Boulenger), *S. monticola*, *S. modesta*, and *S. reevesii* remain poorly understood. Furthermore, molecular data are still unavailable for several poorly known taxa, including *S. barbouri*, *S. doriae*, *S. rara* (Darevsky & Orlov), and *S. schmidti*. These knowledge gaps highlight the need for continued taxonomic and phylogenetic research on this morphologically conserved yet underestimated genus.

During recent herpetological surveys conducted in Qixingguan District, Bijie City, Guizhou Province, China, we collected eight specimens of skinks. Subsequent morphological comparisons and molecular analyses revealed that these specimens belong to the genus *Scincella* but are clearly distinguishable from all known congeners. Accordingly, we describe this previously overlooked population as a new species of *Scincella*, based on an integrative taxonomic approach combining morphological and genetic data.

## ﻿Materials and methods

### ﻿Sampling

**Figure 1.**
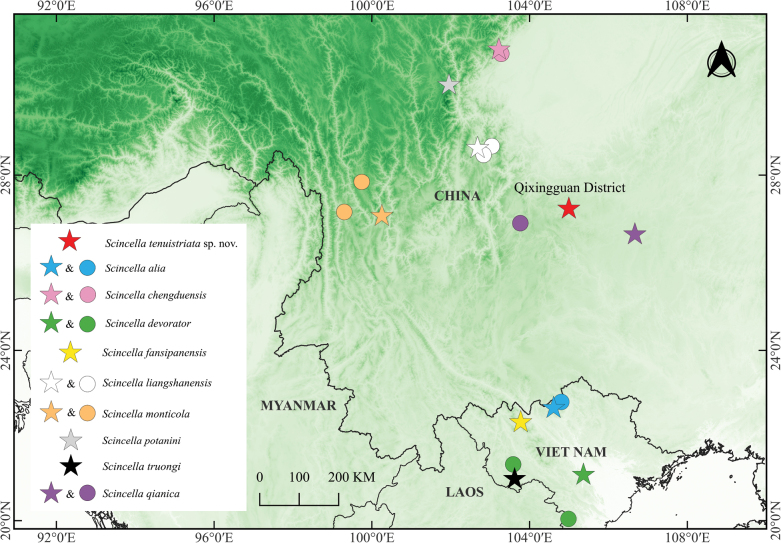
Distribution of *Scincella tenuistriata* sp. nov. and its closest relatives. Stars indicate the type localities, and circles indicate the other known localities. Red star: *Scincella tenuistriata* sp. nov.; blue star and circle: *S. alia*; pink star and circle: *S. chengduensis*; yellow star: *S. fansipanensis*; white star and circles: *S. liangshanensis*; orange star and circles: *S. monticola*; grey star: *S. potanini*; black star: *S. truongi*; and purple star and circle: *S. qianica*.

Eight specimens of *Scincella* sp. were collected in Qixingguan District, Bijie City, Guizhou Province, China (Fig. 1). All newly collected specimens were humanely euthanized using a lethal injection of 0.7% tricaine methanesulfonate (MS222) solution, then fixed and stored in 75% ethanol for long-term preservation. Fresh liver tissue was extracted and immediately preserved in 95% ethanol, and subsequently stored at –20 °C. All specimens were deposited in herpetological collection of the museum of Qinghai University, Qinghai Province, China (**QHU**). The related procedures complied with the Wildlife Protection Law of China and were approved by the Institutional Ethics Committee of Qinghai University (protocol number PJ202501-89).

### ﻿Molecular phylogeny

Since all specimens were collected from the same locality and show consistent morphological traits, we randomly selected five individuals for DNA sequencing. Total genomic DNA was extracted from preserved liver tissue using the QIAamp DNA Mini Kit (QIAGEN, Changsheng Biotechnology Co. Ltd). Three mitochondrial gene fragments were amplified: 12S ribosomal RNA (*12S*) using the primer pair L1091-F (5’-AAACTGGGATTAGATACCCCACTAT-3’) and H1478-R (5’-GAGGGTGACGGGCGGTGTGT-3’) (Kocher et al. 1989); 16S ribosomal RNA (*16S*) using 16SL-F (5’-TGTTTACCAAAAACATAGCCTTTAGC-3’) and 16SL-R (5’-TAGATAGAAACCGACCTGGATT-3’) (Linkem et al. 2011); and cytochrome c oxidase subunit I (*CO1*) using RepCOI-F (5’-TNTTMTCAACNAACCACAAAGA-3’) and RepCOI-R (5’-ACTTCTGGRTGKCCAAARAATCA-3’) (Nagy et al. 2012). The polymerase chain reaction (PCR) was performed in 25 μl reactions with the following cycling conditions: first an initial denaturing step at 95 °C for 5 min; then 35 cycles of denaturing at 95 °C for 40 s, annealing at 53 °C for 40 s and extending at 72 °C for 60 s; last a final extending step at 72 °C for 10 min. The PCR products were sequenced by Shanghai Map Biotech Co., Ltd. Raw sequences were assembled using SeqMan in the DNASTAR software package (Burland 2000). All resulting sequences have been deposited in GenBank under the accession numbers: PV640454–PV640458 (*12S*), PV640464–PV640468 (*16S*), and PV640489–PV640493 (*CO1*) (Table 1).

**Table 1.** GenBank accession numbers, localities, and voucher information for all specimens used in this study.

	Species name	Locality	Voucher NO.	*12S*	*16S*	*CO1*	References
1	*Scincella tenuistriata* sp. nov.	Qixingguan, Bijie, Guizhou, China	QHU R2025008	PV640454	PV640464	PV640489	This study
2	*Scincella tenuistriata* sp. nov.	Qixingguan, Bijie, Guizhou, China	QHU R2025009	PV640455	PV640465	PV640490	This study
3	*Scincella tenuistriata* sp. nov.	Qixingguan, Bijie, Guizhou, China	QHU R2025010	PV640456	PV640466	PV640491	This study
4	*Scincella tenuistriata* sp. nov.	Qixingguan, Bijie, Guizhou, China	QHU R2025011	PV640457	PV640467	PV640492	This study
5	*Scincella tenuistriata* sp. nov.	Qixingguan, Bijie, Guizhou, China	QHU R2025012	PV640458	PV640468	PV640493	This study
6	*S. alia*	Mt. Tay Con Linh, Tuyen Quang, Vietnam	VRTC NAP14081	–	–	PV085567	Bragin et al. (2025a)
7	*S. alia*	Mt. Tay Con Linh, Tuyen Quang, Vietnam	ZMMU Re-18153	PV088911	PV088913	PV085569	Bragin et al. (2025a)
8	*S. assata*	Finca El Milagro, Santa Ana, El Salvador	KU 289795	JF497946	JF498074	–	Linkem et al. (2011)
9	*S. assata*	Canton El Volcan, San Miguel, El Salvador	KU 291286	–	JF498075	–	Linkem et al. (2011)
10	*S. auranticaudata*	Ta Kou NR, Lam Dong, Vietnam	ITBCZ 6527	–	–	PV022548	Nguyen et al. (2025)
11	*S. auranticaudata*	Ta Kou NR, Lam Dong, Vietnam	ITBCZ 7620	–	–	PV022549	Nguyen et al. (2025)
12	*S. badenensis*	Mt. Ba Den, Tay Ninh, Vietnam	ITBCZ 5966	–	–	MK990602	Nguyen et al. (2019)
13	*S. badenensis*	Mt. Ba Den, Tay Ninh, Vietnam	ITBCZ 5993	–	–	MK990603	Nguyen et al. (2019)
14	*S. balluca*	Bidoup-Nui Ba NP, Lam Dong, Vietnam	ZMMU R-13268-NAP-00412	–	–	MH119616	Bragin et al. (2025b)
15	*S. balluca*	Bidoup-Nui Ba NP, Lam Dong, Vietnam	ZMMU R-13268-NAP-01062	–	–	MH119617	Bragin et al. (2025b)
16	*S. baraensis*	Mt. Ba Ra, Dong Nai, Vietnam	ITBCZ 6534	–	–	MT742256	Nguyen et al. (2020)
17	*S. baraensis*	Mt. Ba Ra, Dong Nai, Vietnam	ITBCZ 6536	–	–	MT742258	Nguyen et al. (2020)
18	*S. boettgeri*	Yaeyama Group, Ryukyus, Japan	KUZ R68001	–	–	LC630768	Koizumi et al. (2022)
19	*S. boettgeri*	Yaeyama Group, Ryukyus, Japan	KUZ R68008	–	–	LC630770	Koizumi et al. (2022)
20	*S. chengduensis*	Dayi, Sichuan, China	CIB 107637	PQ466924	PQ466921	PQ467109	Jia et al. (2025)
21	*S. chengduensis*	Chongzhou, Sichuan, China	CIB 118786	PQ466923	PQ466920	PQ467108	Jia et al. (2025)
22	*S. cherriei*	Montes Azules Biosphere Reserve, Chiapas, Mexico	RCMX 219	–	MW265931	–	Castiglia et al. (2020)
23	*S. cherriei*	Montes Azules Biosphere Reserve, Chiapas, Mexico	RCMX 235	–	MW265932	–	Castiglia et al. (2020)
24	*S. devorator*	Ba Vi NP, Ha Noi, Vietnam	ZMMU NAP07169	PV088910	PV088912	PV085573	Bragin et al. (2025a)
25	*S. dunan*	Yonagunijima Is., Southern Ryukyus, Japan	KUZ R65170	–	–	LC630778	Koizumi et al. (2022)
26	*S. dunan*	Yonagunijima Is., Southern Ryukyus, Japan	KUZ R67027	–	–	LC630779	Koizumi et al. (2022)
27	*S. fansipanensis*	Mt. Fansipan, Lao Cai, Vietnam	IEBR R.5185	–	–	LC846671	Okabe et al. (2024)
28	*S. fansipanensis*	Mt. Fansipan, Lao Cai, Vietnam	IEBR R.5187	–	–	LC846672	Okabe et al. (2024)
29	*S. formosensis*	Taiwan, China	KUZ R37515	–	–	LC630789	Koizumi et al. (2022)
30	*S. formosensis*	Taiwan, China	KUZ R37516	–	–	LC630790	Koizumi et al. (2022)
31	*S. gemmingeri*	Teocelo, Mexico	LSUMZ H-14810	AY308294	AY308445	–	–
32	*S. honbaensis*	Hon Ba NR, Khanh Hoa, Vietnam	ITBCZ 4679	–	–	PV022547	Nguyen et al. (2025)
33	*S. huanrenensis*	Pyeongchanggun, Gangwondo, Korea	G390SH	KU507306	KU507306	KU507306	Park et al. (2016)
34	*S. huanrenensis*	Pyeongchanggun, Gangwondo, Korea	–	NC030779	NC030779	NC030779	Park et al. (2016)
35	*S. lateralis*	Texas, USA	DCC 2842	HM852476	HM852503	–	Reeder and Reichert (2011)
36	*S. lateralis*	Texas, USA	KU 289460	JF497948	JF498077	–	Linkem et al. (2011)
37	*S. liangshanensis*	Meigu, Sichuan, China	CIB 119513	PP826317	PP826315	PP824806	Jia et al. (2024)
38	*S. liangshanensis*	Meigu, Sichuan, China	CIB 119514	PP826318	PP826314	PP824804	Jia et al. (2024)
39	*S. liangshanensis*	Yuexi, Sichuan, China	XM-YXS80	PP826316	PP826313	PP824805	Jia et al. (2024)
40	*S. melanosticta*	Kon Chu Rang NR, Gia Lai, Vietnam	ZMMU NAP05519	–	–	MH119621	Neang et al. (2018)
41	*S. melanosticta*	Kon Chu Rang NR, Gia Lai, Vietnam	ZMMU NAP06376	–	–	MH119622	Neang et al. (2018)
42	*S. modesta*	Ningbo, Zhejiang, China	CIB 121415	PP819198	PP819195	PP819217	Jia et al. (2024)
43	*S. modesta*	Ningbo, Zhejiang, China	WYF 11520	PP819197	–	PP819215	Jia et al. (2024)
44	*S. monticola*	ShangriLa, Yunnan, China	DLYNJC 2020824	OP955952	OP955962	–	Jia et al. (2023)
45	*S. nigrofasciata*	Keo Seima WS, Mondulkiri, Cambodia	CBC 2545	–	–	MH119613	Neang et al. (2018)
46	*S. nigrofasciata*	Dak Nong UNESCO Global GeoparkNR, Lam Dong, Vietnam	ITBCZ 11028	–	–	PQ634873	Nguyen et al. (2024)
47	*S. ouboteri*	Ngoc Son-Ngo Luong NR, Phu Tho, Vietnam	IEBR R.5042	–	–	OP927026	Pham et al. (2024)
48	*S. ouboteri*	Ngoc Son-Ngo Luong NR, Phu Tho, Vietnam	IEBR R.5043	–	–	OP927027	Pham et al. (2024)
49	*S. ochracea*	Sop Cop NR, Son La, Vietnam	TBU PAT.254	–	–	OP927028	Pham et al. (2024)
50	*S. potanini*	Kangding, Sichuan, China	DL KD202109071	OP942203	OP935937	OP942210	Jia et al. (2023)
51	*S. potanini*	Kangding, Sichuan, China	DL KD202109072	OP942208	OP935987	OP942209	Jia et al. (2023)
52	*S. reevesii*	Zhaoqing, Guangdong, China	NB 2017030715	NC054206	NC054206	NC054206	Zhong et al. (2021)
53	*S. reevesii*	Zhaoqing, Guangdong, China	–	MN832615	MN832615	MN832615	Zhong et al. (2021)
54	*S. qianica*	Guiyang, Guizhou, China	QHU R2025001	PV527316	PV527321	PV527759	This study
55	*S. qianica*	Guiyang, Guizhou, China	QHU R2025002	PV527317	PV527322	PV527760	This study
56	S. cf. rufocaudata	Ke Go NR, Ha Tinh, Vietnam	ZFMK 76238	–	HM773216	–	Nguyen et al. (2011)
57	S. cf. rufocaudata	Ke Go NR, Ha Tinh, Vietnam	ZFMK 76239	–	HM773217	–	Nguyen et al. (2011)
58	*S. rupicola*	Thailand	KUZ 40458	AB057388	AB057403	–	Honda et al. (2003)
59	*S. vandenburghi*	Tsushima Island, Japan	KUZ R66394	–	–	LC507695	Koizumi et al. (2021)
60	*S. vandenburghi*	Yeongwolgun, Korea	G389SV	KU646826	KU646826	KU646826	Park et al. (2016)
61	*S. wangyuezhaoi*	Wenchuan, Sichuan, China	CIB 87246	OP942191	OP941172	OQ402205	Jia et al. (2023)
62	*S. wangyuezhaoi*	Lixian, Sichuan, China	CIB 119510	OP942192	OP941174	–	Jia et al. (2023)
**Out group**							
63	*Sphenomorphus cryptotis*	Shangsi, Guangxi, China	CIB 119027	OP942206	OP942190	OP942215	Jia et al. (2023)

In addition to newly obtained sequences, we included 100 sequences from 57 individuals representing 31 nominal *Scincella* species (Table 1), and selected *Sphenomorphus cryptotis* Darevsky, Orlov & Cuc as the outgroup (following Jia et al. 2023). Three gene fragments, comprising 384 base pairs (bp) of *12S*, 542 bp of *16S*, and 659 bp of *CO1*, were concatenated into a combined dataset (comprising 1585 bp in total). Sequence alignment was performed using MUSCLE (Edgar 2004) in MEGA X (Kumar et al. 2018). The Maximum Likelihood (ML) analysis was conducted in IQ-TREE v. 1.6.12 (Nguyen et al. 2015) using the best-fit model GTR + F + I + G4 for all three fragments (*12S*, *16S*, and *CO1*), as determined by ModelFinder for IQ-TREE in PhyloSuite v. 1.2.3 according to Bayesian Information Criterion (BIC) (Kalyaanamoorthy et al. 2017; Zhang et al. 2020). Nodes were considered well supported when the ultrafast bootstrap values (UFB) were above 95% and SH-like approximate likelihood ratio test values (SH-aLRT) were above 80% (Stephane et al. 2010; Hoang et al. 2018). The resulting phylogenetic tree was visualized in FigTree v. 1.4.4 (Rambaut 2018). Pairwise genetic distances (*p*-distances) for the *CO1* gene between closely related species were also calculated in MEGA X using the uncorrected distance model.

### ﻿Morphological analyses

Morphological data, including both meristic and morphometric characters, were described following the methodology of Bragin et al. (2025a), with certain character abbreviations revised according to the standards of Darko et al. (2022). Three morphometric characters were measured with Deli digital calipers (No. 90150B) to the nearest 0.1 mm: **SVL** = snout-vent length, measured from the tip of the snout to the posterior edge of vent; **TAL** = tail length, measured from the posterior margin of vent to the tip of tail; **AGD** = axilla-groin distance, defined as the distance between the posterior edge of the forelimb insertion and the anterior edge of the hindlimb insertion, with both limbs inserted perpendicularly to the body wall. All other measurements were taken using Mitutoyo digital calipers (CD-15AX) to the nearest 0.01 mm under Leica stereomicroscope (EZ4): **HL** = head length, measured from the tip of the snout to the caudal extremity of the retroarticular process of the mandible (a prominent point externally visible at the posterior end of the lower jaw); **HW** = head width, the widest portion between the left and right articulations of jaw; **HH** = head height, the deepest portion from ventral to dorsal surface of head; **ED** = eye diameter, the length of the palpebral cleft; **EN** = eye-narial distance, measured from the anterior corner of the eye to the posterior margin of the naris; **PDD** = palpebral disc diameter, the maximum horizontal diameter of palpebral disc; **ESD** = snout length, measured from the tip of the snout to the anterior corner of eye; **EL** = ear opening diameter, maximum diameter of tympanum; **FLL** = forelimb length, measured from anterior junction of forelimb and body wall to tip of the finger IV; **F4L** = finger IV length, measured from the junction between the skin of third and fourth fingers to tip of finger IV; **HLL** = hind-limb length, measured from anterior junction of hindlimb and body wall to tip of toe IV; **T4L** = toe IV length, measured from the junction between the skin of third and fourth toes to tip of toe IV; and **Limbs adpressed** = whether the forelimbs and hindlimbs can make contact when the body is held straight and the limbs are adpressed.

Scalation features and their abbreviations were as follows: **SL** = supralabials; **IL** = infralabials; **FrN** = frontonasals; **SCI** = superciliaries; **SO** = supraoculars; **PF** = prefrontals; **FrP** = frontoparietals; **P** = parietals; **TEMP** = enlarged temporals; **Lor** = loreals; **NU** = nuchals; chin-shields; **GS** = gulars scale; **MBSR** = midbody scale rows, number of longitudinal scale rows measured around the widest point of midbody; **PVSR** = paravertebral scale rows, the number of scale rows counted between parietals and the just posterior margin of hindlimbs; **DBR** = dorsal scale rows between dorsolateral stripes, the number of midbody dorsal scale rows between dark dorsolateral stripes; **SRB** = scale rows covered by dorsolateral stripes; **VS** = ventral scale rows, the number of scale rows counted between gulars and precloacals; **F4S** = number of enlarged, subdigital lamellae beneath finger IV; and **T4S** = number of enlarged, subdigital lamellae beneath toe IV. Sex was determined by dissection, based on the presence of testes or ovaries.

In addition, following Jia et al. (2024), we also examined the diagnostic coloration characters in *Scincella* species: **UMLLS** = the upper margin of dorsolateral stripes wavy or relatively straight, and **VDM** = ventral dark markings, presence or absence of dark-colored markings on the ventral surface.

The comparison with other species of the genus *Scincella* was based on available literature: Gray (1838), Boulenger (1887), Günther (1896), van Denburgh (1912), Stejneger (1925), Barbour (1927), Schmidt (1927), Smith (1935), Taylor (1963), Zhao and Huang (1982), Darevsky and Nguyen (1983), Ouboter (1986), Wang and Zhao (1986), Inger et al. (1990), Darevsky and Orlov (1997), Zhao et al. (1999), Chen et al. (2001), Darevsky et al. (2004), Bourret (2009), Nguyen et al. (2010a, b, 2011, 2019, 2020, 2025), Neang et al. (2018), Koizumi et al. (2022), Jia et al. (2023, 2024), Okabe et al. (2024), Pham et al. (2024), Bragin et al. (2025a, b), Jia et al. (2025), Pham et al. (2025), Xu et al. (2025a, b).

Other abbreviations are as follows: **Is**: Island; **Mt.**: Mountain; **NP**: National Park; **NR**: Nature reserve; **WS**: Wildlife Sanctuary.

### ﻿Statistical analysis

To compare quantitative variation among *Scincella* sp. from Qixingguan District, Bijie City, Guizhou, and its two closely related congeners *S. alia* and *S. qianica* (as suggested by DNA data), we conducted a series of univariate and multivariate statistical analyses to test whether the three species-level lineages occupied distinct morphological clusters and whether they differed significantly from one another.

Prior to statistical analyses, specimens of *Scincella* sp. from Bijie, *S. alia*, and *S. qianica* were sorted based on external morphology and geographic distribution. Specimens with broken tails or incomplete morphological data were excluded from multivariate analyses but retained for univariate comparisons.

Each morphological character was first tested for normality (Shapiro-Wilks test) and homogeneity of variances (Levene’s test). Independent-sample Student’s *t*-tests were conducted to assess sexual dimorphism across the dataset. Since most characters satisfied these assumptions and no significant sexual dimorphism was detected, we pooled males and females (including juveniles) for all subsequent analyses to maximize sample size.

We then applied one-way ANOVA followed by Tukey’s HSD post hoc tests to evaluate pairwise differences in individual morphological traits among the three lineages. For multivariate analyses, eight candidate morphological characters were combined, and a Principal Component Analysis (PCA) was performed on twenty-four specimens (8 *Scincella* sp. from Bijie, 12 *S. alia*, and 4 *S. qianica*). All statistical analyses were conducted in R v.4.4.0 (R Core Team 2024), and PCA visualization was generated using the R package ggplot2 (Wickham 2016). We considered all differences statistically significant at *P* ≤ 0.05.

## ﻿Results

### ﻿Phylogenetic analysis

**Figure 2.**
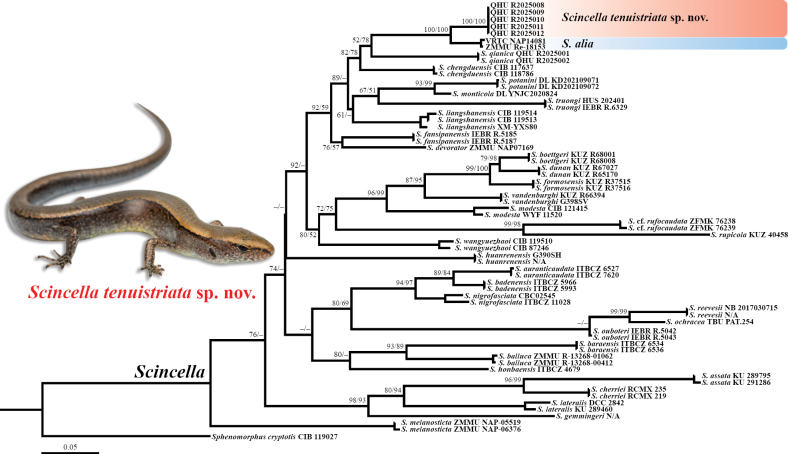
Phylogenetic topology of the genus *Scincella* inferred from three mitochondrial (*12S/16S*/*CO1*) fragments. The nodes supporting values on branches are presented with the SH-like approximate likelihood ratio test (SH) / Ultrafast Bootstrap Approximation (UFB); the ones lower than 50 are displayed as “–”. Photos on thumbnails by YHX.

The topology obtained from the ML analysis is shown in Fig. 2. The phylogenetic reconstruction based on the *12S*, *16S*, and *CO1* genes was largely consistent with previous studies regarding the relationships among species within the major clades of *Scincella* (Jia et al. 2023, 2024, 2025; Bragin et al. 2025a, b). All *Scincella* specimens clustered into a monophyletic group. The specimens from Qixingguan District, Bijie City, Guizhou Province, China formed a strongly supported lineage (SH = 100 / UFB = 100), with nearly no detectable molecular divergence in *CO1* (mean *p*-distance = 0%), and were recovered as the sister group to *S. alia*. The combined clade of the above-mentioned was subsequently grouped with *S. qianica*, but the nodal support was relatively low (SH < 50 / UFB = 78).

The uncorrected *p*-distance are presented in Table 2. Among *Scincella* species, interspecific *p*-distances based on the *CO1* gene ranged from 8.0% (between *S. boettgeri* (Van Denburgh, 1912) and *S. dunan* Koizumi, Ota & Hikida) to 28.7% (between *S. modesta* and *S. ochracea* (Bourret)). In comparison, the newly collected specimens exhibited genetic distances ranging from 8.6% (vs *S. alia*) to 27.2% (vs *S. reevesii*), further supporting their distinctiveness from congeners.

**Table 2.** Uncorrected *p*-distance (%) among the genus *Scincella* species based on partial mitochondria *CO1* gene.

No.	Species	1	2	3	4	5	6	7	8	9	10	11	12	13	14	15	16	17	18	19	20	21	22	23	24	25
1	*S. tenuistriata* sp. nov.	0																								
2	*S. alia*	8.6–8.8	0.8																							
3	*S. auranticaudata*	23.2–23.4	20.9–21.6	0.5																						
4	*S. badenensis*	23	21.3–21.9	9.9–10.0	0																					
5	*S. baraensis*	25.1–25.4	24.7–25.9	22.3–22.8	22.0–22.2	0.2																				
6	*S. boettgeri*	23	20.2–20.8	24.2–24.5	23.4	24.0–24.2	0.2																			
7	*S. chengduensis*	17.4–17.8	16.4–17.0	22.4–22.6	22.0–22.5	25.5–26.2	20.9–21.3	0.3																		
8	*S. devorator*	20.8	19.9–20.1	19.0–19.2	21.3	22.5	20.6	17.3–17.8	–																	
9	*S. balluca*	20.7–20.9	19.5–20.1	21.4–22.0	21.9–22.2	19.1–19.3	21.8–22.0	22.7–23.4	22.1–22.3	1																
10	*S. dunan*	25.1–25.3	22.3–23.2	24.4–24.9	23.5–23.8	26.3–26.7	8.0–8.4	21.1–21.7	20.8–21.0	23.5–23.8	0.2															
11	*S. fansipanensis*	19.1	17.5–18.3	17.5–17.8	19.6	22.1–22.6	20.3	17.9–18.5	15.8	21.2–21.4	22.2–22.5	0														
12	*S. formosensis*	22.9	21.7–22.0	23.5–23.7	24.4	23.4–23.8	9.5	21.3	20.9	23.0–23.1	10.0–10.4	22.9	0.2													
13	*S. honbaensis*	21.4	21.1–22.0	23.4–23.7	23	23	22.5–22.7	20.9–21.4	21.9	19.8–20.4	21.1–21.3	21	23.5	–												
14	*S. huanrenensis*	25.9	22.9–23.8	19.9–20.2	20.8	21.4–21.6	22.1	20.0–20.4	20.8	23.1–23.3	22.1–22.4	19.8–20.0	21.1–21.4	21.8	0											
15	*S. liangshanensis*	18.1–18.7	18.1–19.2	22.5–23.6	21.2–21.3	21.1–22.0	20.2–21.4	17.5–18.5	17.4–17.8	19.6–20.4	23.2–24.3	13.3–14.2	23.2–23.5	18.6–19.7	18.8–20.0	1.1–1.4										
16	*S. melanosticta*	23.2–23.4	21.8–22.3	21.4–22.2	23.1–23.3	22.8	24.7–25.4	22.8–23.4	22.9–23.5	22.3–23.0	26.2–26.9	22.0–22.9	25.4–25.6	22.7	24.0–24.5	21.3–22.3	0.8									
17	*S. modesta*	21.0–22.3	21.8–22.1	22.0–23.1	23.3–23.6	22.6–23.3	17.6–18.1	20.4–22.6	19.7–19.9	18.7–20.9	17.5–20.0	18.4–18.9	19.3–19.9	22.2–22.7	21.6–23.0	19.2–20.5	24.1–24.5	5.5								
18	*S. nigrofasciata*	19.5–19.9	19.8–20.4	13.2–15.0	11.9–12.0	22.9–23.1	23.8–24.1	20.8–22.1	21.9–23.6	20.7–21.6	24.2–25.0	18.0–19.8	24.7–25.0	20.5–21.5	21.2–23.8	20.7–21.7	23.3–24.2	22.4–23.8	5.3							
19	*S. ochracea*	26.8	26.2–26.7	24.1–24.3	24.6	25.1–25.4	24.7–25.0	24.9	24.8	27.4–28.6	24.4–24.7	25.7	24.1	24.8	25.6	23.7–24.3	25.8–26.7	26.8–28.7	24.3–24.4	–						
20	*S. ouboteri*	25.6–26.1	25.2–26.5	22.5–22.9	23.3–23.7	24.0–24.5	22.4–23.1	24.0–24.5	24.7–24.9	25.2–26.7	24.6–25.3	23.3–23.6	22.0–22.4	22.4–22.6	24.2–24.3	23.7–24.6	26.8–27.3	26.5–27.5	23.0–23.8	8.9–9.2	0.3					
21	*S. potanini*	19.8–20.3	19.8–21.1	22.1–22.3	21.8–22.0	22.1–22.8	18.6–19.0	18.8–19.5	16.4–16.9	19.6–20.2	21.9–22.4	16.5–16.8	22.5–22.8	21.7–22.2	20.8–21.3	15.4–16.5	22.4–23.6	19.8–20.8	19.7–20.7	25.6–25.9	24.4–24.9	0.3				
22	*S. qianica*	19.5	28.1–28.3	22.2–22.8	21.6	24.2–24.4	23.4	18.2–18.6	17.9	23.4	24.3–24.5	18.7–18.9	23.7	22.3	23.1	16.0–16.5	23.5–24.1	22.1–24.3	23.8–24.4	26.9	26.0–26.2	19.7–20.0	0			
23	*S. reevesii*	27.2	27.7–27.8	24.8–25.0	24.9	24.6–24.8	24.4–24.6	26.1–26.6	24.5	27.3–27.6	24.8–25.0	25.8–25.9	25.2	25.1	26.1	24.6–25.3	28	25.2–26.6	25.7–25.9	10.2	10.7–11.1	23.5–24.1	26.7	0		
24	*S. truongi*	20.4	19.3–19.5	20.1–20.8	20.8	25.3–25.5	20.7	18.4–18.8	20.7	21.7–22.2	22.6–22.8	18.8	21.7	20.5	24.2	21.1–22.0	26.6–26.8	19.5–20.8	22.7–23.2	27.6	26.0–26.5	21.8–22.3	20.9	27.8	0	
25	*S. vandenburghi*	20.8–21.0	20.6–21.9	23.1–23.5	25.5–25.7	24.3–24.8	15.2–15.4	18.4–19.0	21.2–21.4	19.7–20.6	16.2–16.6	20.1–20.5	16.2–16.4	23.1–23.3	23.1–23.3	19.4–20.5	27.2–27.5	17.4–18.1	22.4–23.8	26.3–26.5	26.1–26.8	20.2–20.7	23.0–23.3	26.2–26.5	20.3–20.5	0.2
26	*S. wangyuezhaoi*	20.7	21.2–22.3	21.3–21.5	21.5	25.3–25.5	24.8	20.4–20.8	20.3	22.8–23.1	23.8–24.0	18.2–18.7	24	23.3	19.2	19.4	24.4–24.7	17.5–21.0	20.4–20.7	26.7	26.1–26.3	19.1–19.6	18.7	27	23.5	19.8–20.0

In light of the well-supported monophyly and significant genetic divergence of the Guizhou populations of *Scincella* sp., along with the unique geographical distribution and a combination of morphological characters, which concordantly differ this population from all known congeners, we herein describe the *Scincella* sp. population from Qixingguan District, Guizhou Province, as a new species.

### ﻿Morphological analysis

**Figure 3.**
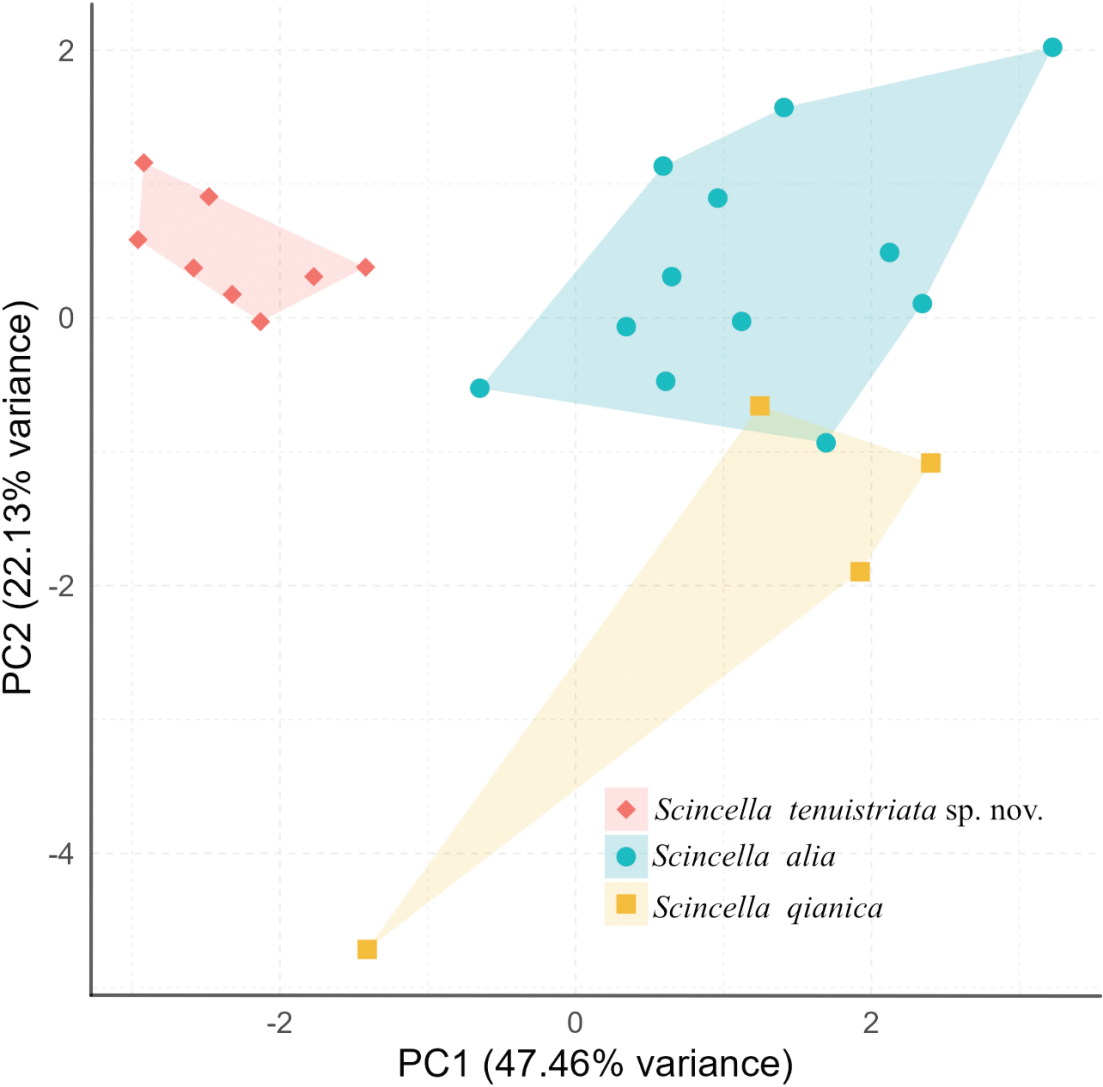
PCA scatter plots of *Scincella tenuistriata* sp. nov., *S. alia*, and *S. qianica*. The shape and color indicated signify different clusters of each *Scincella* spp.

The PCA of the three lineages based on eight morphological characters is shown in Fig. 3. The first two principal components together explained 69.59% of the total variance, with PC1 accounting for 47.46% and PC2 for 22.13%.

PC1 was most strongly loaded on ventral scale counts (VS, loading = 0.4370) and midbody scale rows (MBSR, 0.4303), followed by AGD (0.3482) and SVL (0.3326). PC2 was heavily loaded on snout-vent length (SVL, 0.4991) and AGD (0.4412), with a strong negative loading on paraventral scale rows (PVSR, –0.4310) (Table 3).

**Table 3.** Summary statistics of the principal components analysis (PCA), showing the highest loadings of each morphological character examined and the proportion of variance of each principal component.

Factor	PC1	PC2	PC3	PC4
SVL (mm)	0.3326	0.4991	-0.3566	0.0074
AGD (mm)	0.3482	0.4412	-0.4158	0.1542
MBSR	0.4303	-0.1795	0.1107	-0.4693
PVSR	0.2785	-0.4310	-0.0456	0.744
VS	0.4370	-0.1664	0.0079	0.0462
GS+VS	0.4140	-0.0274	0.3479	0.1194
F4S	-0.2646	-0.3201	-0.6642	0.0590
T4S	0.2702	-0.4554	-0.3511	-0.4271
Eigenvalue	3.7971	1.7708	0.8626	0.5846
Cumulative Eigenvalue % Total Variance	47.46	22.13	10.78	7.31
Cumulative % Total Variance	47.46	69.60	80.38	87.69

The scatterplot of PC1 versus PC2 clearly separated the three lineages into distinct morphological clusters, with *Scincella* sp. from Qixingguan, Bijie, Guizhou forming a discrete group relative to *S. alia* and *S. qianica*. These results indicate that the three lineages occupy distinct morphological spaces and are statistically distinguishable from one another.

### ﻿Taxonomic account

#### ﻿ Scincella tenuistriata sp. nov.

Taxon classificationAnimaliaSquamataScincidae

AC10D6FE-1317-5378-95EC-89AA75C1AD49

http://zoobank.org/2DFFBC0D-8418-4FAA-AF48-064E830B4DCB

**Figure 4.**
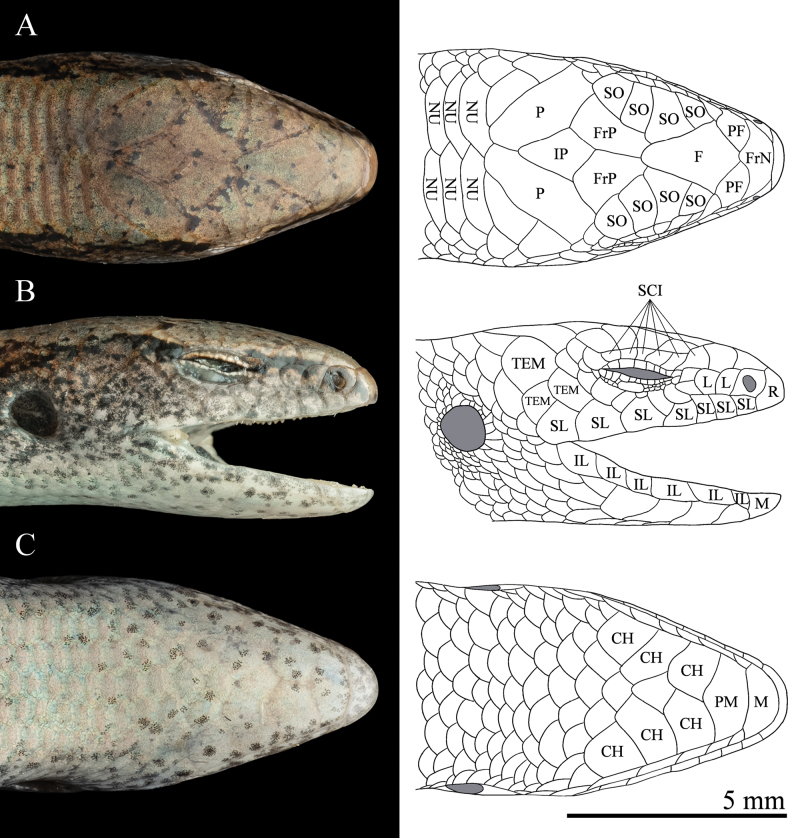
Head scalation of *Scincella tenuistriata* sp. nov. (holotype, QHU R2025009, adult male). A. Dorsal; B. Lateral; C. Ventral views. Notes: CH: chin-shield; F: frontal, FrN: frontonasal; FrP: frontoparietal; IL: infralabials; IP: interparietal; L: loreals; M: mental; NU: nuchals; P: parietal; PF: prefrontal; PM: postmental; R: rostral; SCI: supraciliaries; SL: supralabials; SO: supraoculars; TEM: temporals. Photographs and drawings by YHX. Scale bars: 5 mm.

**Figure 5.**
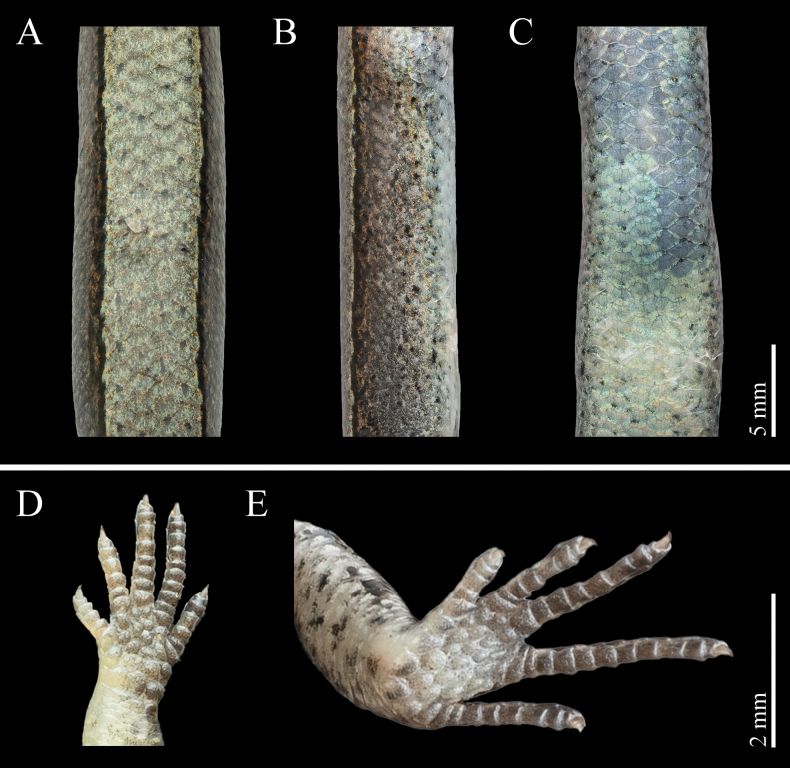
Dorsal (A), lateral (B), and ventral (C) views of the body; D. Ventral view of the hand; E. Ventral view of the foot of *Scincella tenuistriata* sp. nov. (holotype, QHU R2025009, adult male) in preservation. Photographs by YHX. Scale bars: 5 mm (A–C); 2 mm (D, E).

**Figure 6.**
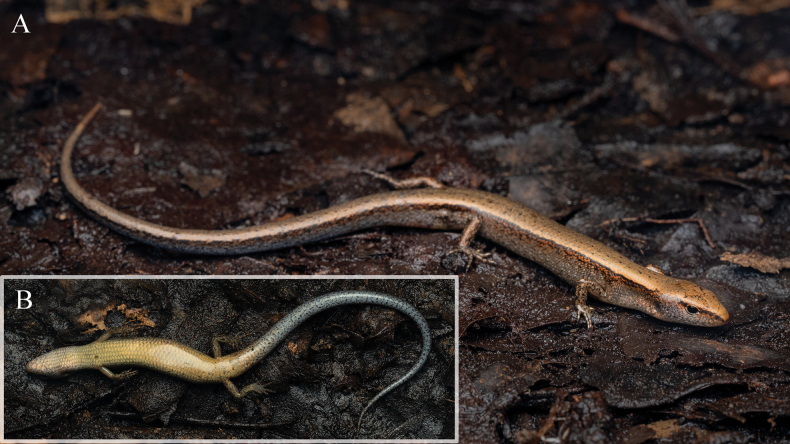
Dorsal (A) and ventral (B) views of the holotype (QHU R2025009, adult male) of *Scincella tenuistriata* sp. nov. in life in situ. Photographs by YHX.

**Figure 7.**
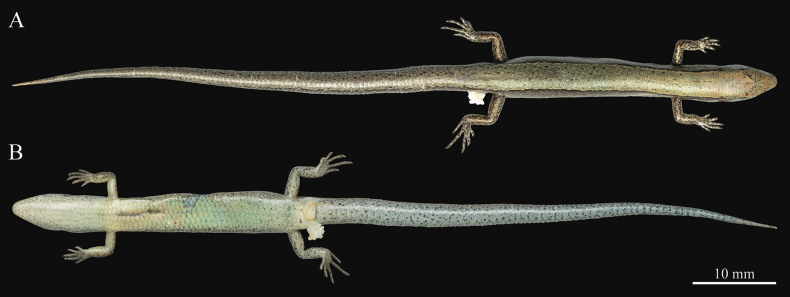
Dorsal (A) and ventral (B) views of the holotype (QHU R2025009, adult male) of *Scincella tenuistriata* sp. nov. in preservation. Photographs by YHX. Scale bars: 10 mm.

**Figure 8.**
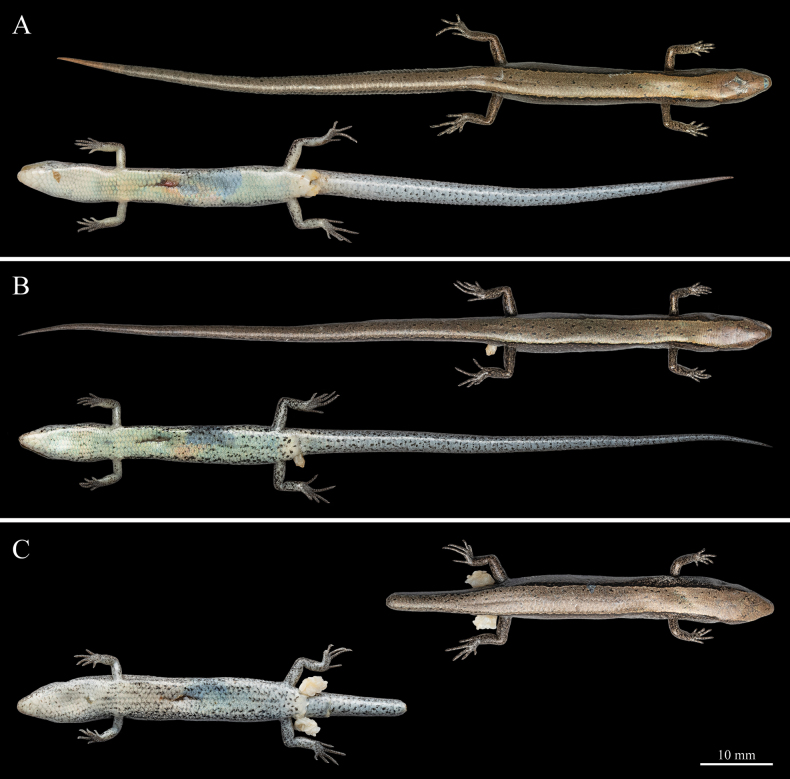
Coloration of paratypes of *Scincella tenuistriata* sp. nov. in preservation. A. QHU R2025010, adult male; B. QHU R2025011, adult male; C. QHU R2025012, adult male. Photographs by YHX. Scale bars: 10 mm.

Tables 4, 5, Figs 4, 5, 6, 7, 8

##### Type material.

***Holotype*.** • QHU R2025009, adult male, from Qixingguan District, Bijie City, Guizhou Province, China (27.2166°N, 105.0015°E; elevation ca. 1,850 m a.s.l.) collected by ZHG on April 15, 2025. ***Paratypes* (*n* = 7)**. • QHU R2025008 and QHU R2025010–015, seven adult males, with the same collecting information as the holotype.

##### Diagnosis.

*Scincella tenuistriata* sp. nov. can be diagnosed from other *Scincella* species by the following unique combination of characters: (1) medium body size in adult male, with a maximum SVL of 42.4 mm; (2) supraciliaries six; (3) supralabials seven, separated from the eye by a row of small scales; (4) infralabials six, rarely five; (5) tympanum deeply recessed and without lobules, with a tympanum diameter significantly larger than the palpebral disc (ear opening diameter / palpebral disc diameter ratio 1.84–2.25; (6) primary temporal single; (7) midbody scale rows 24; (8) ventral scale rows (excluding gulars) 40–43, gulars 21–23, with total ventral + gular scale rows numbering 61–66; (9) toes nearly or just touching fingers when limbs are adpressed; (10) 9 or 10 enlarged lamellae beneath finger IV, and 11 or 12 beneath toe IV; (11) the dark dorsolateral stripes narrow and wavy, covering 0.5–1 scale rows on the trunk, with four scale rows in between on the dorsum; (12) dorsal surface of body brassy, scattered with small dark sports; (13) in life, the ventral surface of the trunk is yellow, scattered with irregular dark spots.

##### Description of the holotype.

Adult male in a good state of preservation with size medium, (SVL 40.3 mm); tail relatively long (TAL 63.7 mm, TAL/SVL ratio 1.58). Axilla-groin distance 22.2 mm, AGD/SVL ratio 0.55. Head elongated, indistinct from the neck (HL 8.41 mm, HW 4.75 mm, HH 3.11 mm). Snout short, obtuse, round anteriorly (ESD 2.31 mm, EN 2.00 mm). Eye large (ED 2.13 mm), lower eyelid with an undivided transparent palpebral disc (window), PDD 0.80 mm. Ear nearly circular; tympanum recessed and distinctly larger than the palpebral disc (EL 1.47 mm, EL/PDD ratio 1.84). Limbs relatively short, toes nearly touching fingers when limbs are adpressed (FLL 9.78 mm, HLL 11.77 mm, F4L 2.11 mm, T4L 3.88 mm, FLL/SVL ratio 0.24, HLL/SVL ratio 0.29). Digits moderately long and slender, each ending in a clearly visible, slightly curved claw. Relative digit lengths of the manus: IV > III > II > V > I, and of the pes: IV > III > V > II > I.

***Head scalation* (Fig. 4).** Head scales smooth. Rrostral convex, wider than high, distinctly visible from above, in contact with the 1^st^ supralabials, nasals, and frontonasal; supranasals absent; frontonasal one, approximately boat-shaped, width ~2× the height., in contact with the rostral, nasals, anterior loreals, prefrontals and frontal; prefrontals two, not in contact with each other, separated medially by frontal; frontal slender, longer than wide, diamond-shaped, in contact with the 1^st^ superciliary, 1^st^ and 2^nd^ supraoculars laterally; a pair of frontoparietals, in contact with each other anteriorly, bordered by frontal, 2^nd^ to 4^th^ supraoculars, interparietal and parietals; interparietal diamond-shaped, width less than height; parietals large, in contact posteriorly, posterolateral border surrounded by the upper secondary temporals, prenuchals, and enlarged nuchals; a single pair of prenuchals, and three pairs of enlarged nuchals.

Nostril oval, located at the center of the nasal; nasal entire, diamond-shaped, width approximately equal to height, in contact with the rostral, frontonasal, 1^st^ loreal, and 1^st^ supralabial; loreals 2, subequal in size; supraoculars 4/4, the 1^st^ contacts the frontal, the 2^nd^ is the largest and contacts both the frontal and frontoparietals, and the 3^rd^ and 4^th^ contact the frontoparietals; superciliaries 6/6, the 1^st^ is the largest; the palpebral disc is bordered by a series of small scales; temporals 1+2, the anterior one subrectangular, the upper secondary temporal is the largest, while the lower one is smaller and broadly contacts the upper; supralabials 7/7, 1^st^ smallest, 5^th^ below the window, 6^th^ largest.

Mental wider than long, round anteriorly, in contact with the 1^st^ infralabials and postmental; postmental large and subpentagonal, contacting the mental, the first two infralabials on each side, and the first pair of chin shields; infralabials 5/6, 1^st^ smallest, 4^th^/6^th^ largest; three pairs of chin shields, the first pair in contact medially, the second pair separated by one gular scale, and the third pair separated by three gulars; gulars 22.

***Body scalation* (Fig. 5).** Body scalation smooth, scales around midbody in 24 rows; distinctly larger than lateral scales and slightly larger than ventrals; paravertebral scale series composed of 51 scales; dorsal scales between dorsolateral stripes 1/2+4+1/2. Ventral scales slightly enlarged medially, decreasing toward the flanks; ventral scale rows (excluding gulars) 41, GS+VS 63; medial pair of precloacal scales enlarged, the left one overlapping the right one. Tail complete; tail scales imbricate and generally uniform in shape, except for the markedly widened subcaudals. Limbs pentadactyl; dorsal surface of fingers and toes covered with two interdigitating scale rows; 10 enlarged lamellae beneath finger IV and 11 beneath toe IV.

***Coloration of the holotype in life* (Fig. 6).** In life, dorsal surface of the head is brassy, scattered with small, irregular dark spots. The upper lateral margins of the head also copper-colored, gradually fading to light brown ventrally, and densely covered with small, ink splatter-like dark spots. The ventral surface of head is creamy white, marked with irregular dark blotches. Each blotch is smaller than a single scale and composed of clusters of over a dozen minute dots.

The dorsal surface of the body and tail is brassy, scattered with small, irregular dark spots. A dark dorsolateral stripe is slightly wavy and very narrow, covering ~0.5–1 scale row on the trunk. The stripe originates at the snout, follows the upper edges of the nasal and loreals, is interrupted at the eye, then resumes posterior the eye and extends along the flanks above the forelimbs and hindlimbs, reaching the tip of the tail. The ground color of the upper flanks is dark brown, bearing scattered black spots that occasionally coalesce into broken, irregular longitudinal streaks. Further ventrally, the brown gradually fades, with the surface marked by small cream and blackish-brown speckles. Near the ventral edge, the brown coloration breaks up further and merges gradually into the lighter ventral coloration. The ventral surface of the trunk is yellow, with a few small, irregular dark spots. The ventral surface of the tail is yellow basally, transitioning to gray posteriorly and densely covered with small dark spots throughout.

***Coloration of the holotype in preservation* (Fig. 7).** After one month in ethanol, the coloration remains similar to that in life, except that the lateral body appears paler, the ventral surface of the trunk faded to a very light cream yellow, and the ventral surface of the tail has turned grayish white.

##### Variation.

Morphometric and meristic data of the type series of *Scincella tenuistriata* sp. nov. are provided in Table 4. The paratypes exhibit coloration generally similar to the holotype, with minor individual variation. Notably, QHU R2025010 has an almost spotless ventral surface at midbody, whereas QHU R2025011, QHU R2025012, and QHU R2025013 bear numerous large, dark spots scattered across the venter (Fig. 8). The main differences in morphometric and scalation characters among the type series (*n* = 8) are as follows: TAL/SVL ratio 1.58–1.75; toes can touch the fingers when limbs are adpressed in specimens QHU R2025010, QHU R2025014, and QHU R2025015; PVSR 51–61; VS 40–43; GS 21–23, GS+VS 61–65; prefrontals in contact in specimens QHU R2025012, QHU R2025013, QHU R2025014, and QHU R2025015; NU 3–4; and F4S 9–10, T4S 11–12.

**Table 4.** Main morphological characteristics of the type series (all males) of *Scincella tenuistriata* sp. nov. All measurements are in mm, the abbreviations of morphological characters are defined in the Materials and methods section.

Specimen	QHU R2025009	QHU R2025008	QHU R2025010	QHU R2025011	QHU R2025012	QHU R2025013	QHU R2025014	QHU R2025015	Range
Type	Holotype	Paratype	Paratype	Paratype	Paratype	Paratype	Paratype	Paratype	
Sex	♂	♂	♂	♂	♂	♂	♂	♂	
Original tail	Yes	No	No	Yes	No	No	Yes	No	
SVL	40.3	42.4	41.0	38.1	40.5	39.6	37.6	40.2	37.6–42.4
TAL	63.7	–	–	66.7	–	–	60.5	–	60.5–66.7
TAL/SVL	1.58	–	–	1.75	–	–	1.61	–	1.58–1.75
AGD	22.2	22.7	22.4	20.8	22.3	22.0	21.8	22.4	21.8–22.7
AGD/SVL	0.55	0.54	0.55	0.55	0.55	0.56	0.58	0.56	0.54–0.58
HL	8.41	9.21	8.17	8.57	8.22	8.60	8.15	8.87	8.15–9.21
HW	4.75	4.95	4.52	4.64	4.76	4.82	4.32	4.91	4.52–4.95
HH	3.11	3.79	3.36	3.15	3.43	3.64	2.91	3.98	2.91–3.98
ED	2.13	2.32	2.09	2.27	2.25	2.09	2.07	2.09	2.07–2.32
ESD	2.31	2.36	2.46	2.33	2.56	2.62	2.73	2.71	2.31–2.73
EN	2.00	1.95	1.82	1.53	1.59	1.86	1.82	1.87	1.53–2.00
PDD	0.80	0.67	0.65	0.74	0.78	0.78	0.63	0.75	0.63–0.80
EL	1.47	1.51	1.33	1.47	1.50	1.52	1.32	1.45	1.33–1.52
EL/PDD	1.84	2.25	2.05	1.99	1.92	1.95	2.10	1.93	1.84–2.25
FLL	9.78	9.66	9.67	9.68	9.01	9.44	9.64	9.77	9.01–9.78
FLL/SVL	0.24	0.23	0.24	0.25	0.22	0.24	0.26	0.24	0.22–0.26
HLL	11.77	12.63	12.94	11.77	12.46	12.33	12.56	13.89	11.77–13.89
HLL/SVL	0.29	0.30	0.32	0.31	0.31	0.31	0.33	0.35	0.29–0.35
F4L	1.80	1.82	2.00	2.12	2.03	2.13	1.93	2.16	1.80–2.16
T4L	3.41	3.56	3.67	3.59	3.07	3.01	3.26	3.49	3.01–3.67
PF	2, separated	2, separated	2, separated	2, separated	2, in contact	2, in contact	2, in contact	2, in contact	separated or in contact
FrP	in contact	in contact	in contact	in contact	in contact	in contact	in contact	in contact	in contact
P	in contact	in contact	in contact	in contact	in contact	in contact	in contact	in contact	in contact
SO	4/4	4/4	4/4	4/4	4/4	4/4	4/4	4/4	4
SCI	6/6	6/6	6/6	6/6	6/6	6/6	6/6	6/6	6
Lor	2/2	2/2	2/2	2/2	2/2	2/2	2/2	2/2	2
TEMP	1+2/1+2	1+2/1+2	1+2/1+2	1+2/1+2	1+2/1+2	1+2/1+2	1+2/1+2	1+2/1+2	1+2
SL	7/7	7/7	7/7	7/7	7/7	7/7	7/7	7/7	7
IL	5/6	6/6	6/6	6/6	6/6	6/6	6/6	6/6	5–6
Chin shields (pair)	3	3	3	3	3	3	3	3	3
NU	3	4	3	4	3	4	4	4	3–4
MBSR	24	24	24	24	24	24	24	24	24
PVSR	51	56	61	56	58	56	57	59	51–61
DBR	1/2+4+1/2	1/2+4+1/2	1/2+4+1/2	1/2+4+1/2	1/2+4+1/2	1/2+4+1/2	1/2+4+1/2	1/2+4+1/2	1/2+4+1/2
GS	22	22	23	21	21	22	21	21	21–23
VS	42	41	43	40	41	43	42	42	40–43
GS+VS	64	63	66	61	62	65	63	63	61–66
F4S	10	10	9	9	10	9	9	10	9–10
T4S	11	11	11	11	11	12	12	11	11–12
SRB	0.5–1	0.5–1	0.5–1	0.5–1	0.5–1	0.5–1	0.5–1	0.5–1	0.5–1
UMLLS	wavy	wavy	wavy	wavy	wavy	wavy	wavy	wavy	wavy
VDM	spots	spots	spots	spots	spots	spots	spots	spots	spots
Limbs adpressed	No	No	Yes	No	No	No	Yes	Yes	Yes or No

##### Distribution and natural history notes.

**Figure 9.**
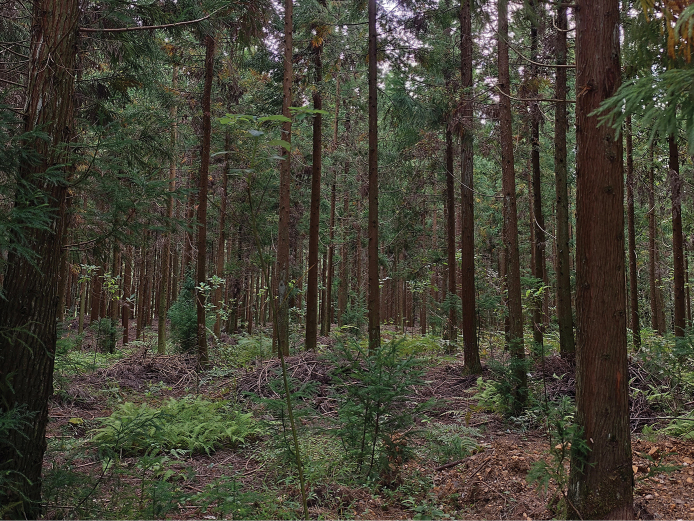
Habitat of *Scincella tenuistriata* sp. nov. in Qixingguan District, Bijie City, Guizhou Province, China.

To date, *Scincella tenuistriata* sp. nov. is currently known only from its type locality. All specimens were collected in April 2025 at elevations ranging from 1,850 m a.s.l., under leaf litter and beneath rocks along a shaded mountain trail. The surrounding habitat is characterized by well-preserved forest dominated by coniferous tree species, with some broad-leaved trees mixed in, indicative of a cool, moist montane environment. (Fig. 9).

During the survey, daytime temperatures averaged ~24 °C, dropping to ~15 °C at night. The skinks were noticeably more active during daylight hours, particularly in the morning and late afternoon, when they were frequently observed actively moving across the forest floor. At night, individuals retreated beneath cover objects and could only be located through careful searching. Fecal analysis revealed small beetle elytra and partially digested crickets, indicating a diet primarily composed of small, non-venomous arthropods, particularly insects.

##### Comparisons.

Based on both morphological and molecular evidence, *Scincella tenuistriata* sp. nov. is closely related to *S. alia*, *S. chengduensis*, *S. devorator* (Darevsky, Orlov & Cuc), *S. fansipanensis*, *S. liangshanensis*, *S. monticola*, *S. potanini*, *S. qianica* and *S. truongi* Pham, Ziegler, Pham, Hoang, Ngo & Le. Detailed morphological comparisons between *Scincella tenuistriata* sp. nov. and its closely related congeners are shown in Table 5.

**Table 5.** Comparisons of main morphological characters between *Scincella tenuistriata* sp. nov. and eight closely related species of *Scincella*.

Species	*S. tenuistriata* sp. nov.	*S. alia*	*S. chengduensis*	*S. fansipanensis*	*S. liangshanensis*	*S. monticola*	*S. potanini*	*S. qianica*	*S. truongi*
SVL (mm)	**37.6–42.**4	38.2–48.2	28.4–43.2	**43.5–59.**0	**43.1–61.9**	36.3–53.0	26.6–57.9	**27.7–44.8**	**49.0–59.4**
TAL (mm)	60.5–66.7	63.2–84.8	59.9	–	54.4–83.8	–	–	49.0–87.9	91.8–100.8
TAL/SVL	**1.58–1.75**	1.5–1.8	1.59	–	0.96–1.71	1.61	**1.02–1.12**	**1.76–2.14**	1.70–1.71
AGD	21.8–22.7	20.6–29.4	/	26.4–35.4	–	–	–	15.6–25.6	25.3–34.3
AGD/SVL	0.54–0.58	0.53–0.63	0.55–0.61	–	0.56–0.66	0.56–0.65	0.52–0.72	0.56–0.59	0.52–0.59
FLL/SVL	**0.22–0.26**	0.20–0.27	0.21–0.26	–	**0.14–0.22**	**0.13–0.19**	0.11–0.25	**0.18–0.21**	0.22–0.27
HLL/SVL	**0.29–0.35**	0.25–0.34	0.23–0.32	–	0.22–0.33	**0.20–0.22**	0.17–0.31	**0.27–0.30**	0.29–0.38
EL/PDD	**1.84–2.25**	1.63–2.18	**1.05–1.58**	–	1.25–2.38	**0.62–1.11**	**0.79–1.25**	**1.65–1.92**	**1.33–1.52**
PF	in contact or separated	in contact or separated	in contact or separated	separated	in contact or separated	in contact	in contact or separated	separated	separated
FrP	in contact	in contact	in contact	in contact	in contact	in contact	in contact	in contact	in contact
P	in contact	in contact	in contact	in contact	in contact or separated	in contact	in contact	in contact	in contact
SO	4	4	4	4	4	4	4	4	4
SCI	6	6	6–7	5–6	6–7	6–7	6–7	6–7	7–8
Lor	2	2	2	2	2	2	2	2	2
TEMP	**1+2**	1+2+2	1+2–2	1+2	1+2–2+3	1+2–2+2	1+2–2+2	1+2	**2+2**
SL	7	7	7	6–7	7	7	7–8	7	7–8
IL	6 (rarely 5)	6	7	6	7	7	7	6–7	6–7
NU	3–4	3–4	3–4	2–6	2–5	3–4	3	3	3
MBSR	**24**	**26–28**	**23**	22–24	23–27	23–25	24–27	**26**	**28**
PVSR	**51**–**61**	56–63	57–60	**60–68**	**69–80**	**62–80**	**69–73**	**61–66**	**60–67**
DBR	**1/2+4+1/2**	1/2+4+1/2	1/2+4+1/2	1/2+4+1/2	1/2+4+1/2	1/2+4+1/2	1/2+4+1/2	1/2+4+1/2	**6**
GS	**21–23**	22–25	21–22	–	22–29	22–24	**23–25**	20–24	–
VS	**40–43**	**44–52**	42–44	–	**43–57**	**45–52**	**45–64**	**46–53**	–
GS+VS	**61–66**	**66–76**	64–65	58–64	**68–82**	**67–77**	**69–89**	**66–75**	**60–70**
F4S	**9–10**	7–10	**8–9**	**7–9**	8–11	8–10	7–10	9–10	10
T4S	11–12	11–13	10–12	10–12	10–15	10–12	10–13	**13–14**	13–15
SRB	**0.5–1**	**1**	**1–2.5**	–	**1.5–2.5**	**1.5–2**	**1.5–3**	**1–1.5**	–
UMLLS	**wavy**	wavy	**straight**	**straight**	**straight**	**straight**	**straight**	**straight**	wavy
VDM	**spots**	**absent**	spots	**absent**	**absent**	**absent**	**absent**	**stripes**	**absent**
Limbs adpressed	Yes or No	No	No	No	No	No	No	No	No

*Scincella tenuistriata* sp. nov. can be distinguished from *S. alia* by MBSR 24 (vs 26–28); VS 40–43 (vs 44–52); GS+VS 61–66 (vs 66–76); and by the presence of dark spots on the middle of the ventral surface (vs absence). It can be distinguished from *S. chengduensis* by EL/PDD 1.84–2.25 (vs 1.05–1.58); F4S 9–10 (vs 8–9); MBSR 24 (vs 23); and dorsolateral stripes being narrow and wavy, covering ~0.5–1 scale rows on the trunk (vs stripes with relatively straight upper margins, covering ~1–2.5 scale rows). It can be distinguished from *S. devorator* by MBSR 24 (vs 30); SCI 6 (vs 8); T4S 11–12 (vs 17); and PVSR 51–61 (vs 68). It can be distinguished from *S. fansipanensis* by smaller body size, SVL 37.6–42.4 mm (vs 43.5–59.0 mm); F4S 9–10 (vs 7–9); PVSR 51–61 (vs 60–68); and presence of dark spots on the ventral surface (vs absence). It can be distinguished from *S. liangshanensis* by smaller body size, SVL 37.6–42.4 mm (vs 43.1–61.9 mm); EL/PDD ratio 1.84–2.25 (vs 0.14–0.22); PVSR 51–61 (vs 69–80); VS 40–43 (vs 43–57); dorsolateral stripes being narrow and wavy, covering ~0.5–1 scale rows on the trunk (vs stripes with relatively straight upper margins, covering ~1.5–2.5 scale rows); and presence of dark spots on the ventral surface (vs absence). It can be distinguished from *S. monticola* by PVSR 51–61 (vs 62–80); VS 40–43 (vs 45–52); (3) GS+VS 61–66 (vs 67–77); FLL/SVL 0.22–0.26 (vs 0.13–0.19); HLL/SVL 0.29–0.35 (vs 0.20–0.22); EL/PDD 1.84–2.25 (vs 0.62–1.11); dorsolateral stripes being narrow and wavy, covering ~0.5–1 scale rows on the trunk (vs stripes with relatively straight upper margins, covering ~1.5–2 scale rows); and presence of dark spots on ventral surface (vs absence). It can be distinguished from *S. potanini* by a comparatively longer tail, TAL/SVL ratio 1.58–1.75 (vs 1.02–1.12); a greater EL/PDD ratio (1.84–2.25 vs 0.79–1.25); PVSR 51–61 (vs 69–73); the dorsolateral stripes narrow and wavy, covering ~0.5–1 scale rows on the trunk (vs stripes with relatively straight upper margins, covering ~1.5–3 scale rows); and presence of dark spots on the ventral surface (vs absence). It can be distinguished from *S. qianica* by MBSR 24 (vs. 26); PVSR 51–61 (vs. 61–66); VS 40–43 (vs. 46–53); GS+VS 61–66 (vs. 66–75); and T4S 11–12 (vs. 13–14) Furthermore, it can be distinguished from *S. truongi* by smaller body size, SVL 37.6–42.4 mm (vs 49.0–59.4 mm); MBSR 24 (vs 28); TEMP 1+2 (vs 2+2); EL/PDD ratio 1.84–2.25 (vs 1.33–1.52); and presence of dark spots on the ventral surface (vs absence).

Among the other three Chinese congeners (including *S. tsinlingensis*, *S. huanrenensis*, *andS. schmidti*) that share the character of having four dorsal scale rows between the dorsolateral stripes, *Scincella tenuistriata* sp. nov. can be distinguished from *S. tsinlingensis* by MBSR 24 (vs 26–28); PVSR 51–61 (vs.70–90); GS+VS 61–66 (vs 83–98); EL/PDD ratio 1.84–2.25 (vs 0.80–1.14); and TAL/SVL ratio 1.58–1.75 (vs 1.24–1.42). It can be distinguished from *S. huanrenensis* by having MBSR 24 (vs 25–28); PVSR 51–61 (vs 66–79); T4S 11–12 (vs 13–16); EL/PDD ratio 1.84–2.25 vs (0.61–1.14); and TAL/SVL ratio 1.58–1.75 (vs 1.19–1.47). And it can be distinguished from *S. schmidti* by having MBSR 24 (vs 26); PVSR 51–61 (vs 68–91); GS+VS 61–66 (vs 71–87); and TAL/SVL ratio 1.58–1.75 (vs 1.9). In comparison with the remaining congeners in China, *Scincella tenuistriata* sp. nov. can be easily distinguished from *S. barbouri*, *S. doriae*, *S. formosensis*, *S. modesta*, *S. przewalskii*, *S. reevesii*, and *S. wangyuezhaoi* by having four dorsal scale rows between the dorsolateral stripes (vs 6–8). Moreover, it can be distinguished from *S. barbouri* by having T4S 11–12 (vs 15–17); from *S. doriae* by having MBSR 24 (vs 30–32), T4S 11–12 (vs 15–18); from *S. formosensis* by having MBSR 24 (vs 28–29), and T4S 11–12 (vs 14–18); from *S. modesta* by having TAL/SVL ratio 1.58–1.78 (vs 1.2–1.4), MBSR 24 (vs 26–28), and T4S 11–12 (vs 13–15); from *S. przewalskii* by having SO 4 (vs 3) and T4S 11–12 (vs 17); from *S. reevesii* by having T4S 11–12 (vs 15–18); and from *S. wangyuezhaoi* by having MBSR 24 (vs 27–30), and VS 40–43 (vs 46–59).

In addition, *Scincella tenuistriata* sp. nov. can be easily distinguished from other Asian congeners as follows: from *S. apraefrontalis* Nguyen, Nguyen, Böhme & Ziegler, *S. auranticaudata* Nguyen, Nguyen, Le, Nguyen, Phan, Vo, Murphy & Che, *S. badenensis* Nguyen, Nguyen, Nguyen & Murphy, *S. balluca* Bragin, Zenin, Le, Nguyen, Nguyen & Poyarkov, *S. baraensis* Nguyen, Nguyen, Nguyen & Murphy, *S. boettgeri*, *S. capitanea* Ouboter, *S. darevskii* Nguyen, Ananjeva, Orlov, Rybaltovsky & Böhme, *S. dunan*, *S. honbaensis* Nguyen, Nguyen, Le, Nguyen, Phan, Vo, Murphy & Che, *S. melanosticta*, *S. nigrofasciata* Neang, Chan & Poyarkov, *S. ochracea*, *S. ouboteri* Pham, Pham, Le, Ngo, Ziegler & Nguyen, *S. rara*, *S. rufocaudata* (Darevsky & Nguyen), *S. rupicola* (Smith), *S. truongi* Pham, Ziegler, Pham, Hoang, Ngo & Le, *S. vandenburghi* (Schmidt), and *S. victoriana* (Shreve) by having 24 MBSR (vs 18 in *S. apraefrontalis*, 34–36 in *S. auranticaudata*, 30–32 in *S. balluca*, 32–36 in *S. badenensis*, 30 in *S. baraensis*, 26–32 in *S. boettgeri*, 30–32 in *S. capitanea*, 28 in *S. darevskii*, 26–29 in *S. dunan*, 28 in *S. honbaensis*, 30–32 in *S. melanosticta*, 32–33 in *S. nigrofasciata*, 30–32 in *S. ochracea*, 30–32 in *S. ouboteri*, 24 in *S. rara*, 30–34 in *S. rufocaudata*, 33–36 in *S. rupicola*, 28–30 in *S. vandenburghi*, and 26 in *S. victoriana*); and from *S. punctatolineata* Boulenger by nuchals present (vs absent) and T4S 11–12 (vs 13–15).

##### Etymology.

The specific name *tenuistriata* is a Latin adjective in the nominative singular (adjusted to the feminine gender of the genus name), derived from the Latin words *tenuis* (meaning narrow) and *stria* (meaning furrow, channel; *striatus* meaning striped). The name is given in reference to the narrow, dark dorsolateral stripes of the new species. We propose the following common names for this species: 细纹滑蜥 (Xì Wén Huá Xī) in Chinese, “Narrow-striped Ground Skink” in English, and “Tonkopolosyi malyi stsink” (Тонкополосый малый сцинк) in Russian.

## ﻿Discussion

In this study, we combined morphological and molecular analyses of *Scincella* ground skinks from Qixingguan District, Bijie City, Guizhou Province, China, to provide robust evidence for the recognition of the new species. Molecular phylogenetic analysis indicated that *Scincella tenuistriata* sp. nov. is most closely related to *S. alia* recently described species from northeastern Vietnam, with an uncorrected *p*-distance of 8.6–8.8% based on the *CO1* gene. However, morphologically, the new species can be readily distinguished from *S. alia* by its fewer midbody and ventral scale rows, and the presence of distinct dark spots on the ventral surface. Moreover, these two species are also geographically separated by a distance of more than 600 km, with the new species occurring in the eastern Wumeng Mountains in China, while *S. alia* currently restricted to the Tay Con Linh Mountain Range in northeastern Vietnam, though its distribution may extend into adjacent areas of China. This clear geographic isolation likely contributed to their genetic and morphological divergence.

*Scincella potanini* and *S. monticola* were historically believed to be widely distributed across the mountainous regions of western China and northern Vietnam (Inger et al. 1990; Zhao et al. 1999; Zhao 2003; Cai et al. 2018; Bragin et al. 2025a). However, with expanded field surveys and the application of DNA barcoding techniques, the taxonomic identities of many populations previously assigned to these species have been gradually re-evaluated. More than half of the *Scincella* species described in the past five years now placed in the *S. potanini*-*S. monticola* complex (e.g., Jia et al. 2023, 2024, 2025; Okabe et al. 2024; Bragin et al. 2025a; Pham et al. 2025). These findings suggest that the actual distributions of *S. potanini* and *S. monticola* sensu stricto are likely far more restricted than previously assumed. The discovery of *Scincella tenuistriata* sp. nov. provides further evidence that the true diversity within the *S. potanini*-*S. monticola* group remains substantially underestimated and underscores the urgent need for acomprehensive taxonomic revision of this complex.

In addition, consistent with most previous studies on *Scincella* taxonomy, the phylogenetic tree presented in this study exhibits generally low support at many of the deeper nodes (Neang et al. 2018; Nguyen et al. 2019, 2020; Koizumi et al. 2022; Jia et al. 2023, 2024; Okabe et al. 2024; Pham et al. 2024, 2025; Bragin et al. 2025a, b; Jia et al. 2025). This result likely reflects two major limitations: the restricted availability of genetic markers primarily mitochondrial DNA and the still insufficient taxon sampling taxon across the genus (Bragin et al. 2025a). Together, these factors underscore the urgent need for broader geographic and taxonomic sampling as well as the incorporation of additional genetic data, particularly nuclear markers, to improve phylogenetic resolution and facilitate more accurate species delimitation within the genus *Scincella*.

The new species *Scincella tenuistriata* sp. nov. inhabits well-preserved coniferous forest at elevations of 1,850 m in the eastern Wumeng Mountains, Guizhou Province. The microhabitat consists mainly of shaded forest floor with abundant leaf litter, stones, and decomposing logs, where individuals were observed foraging during the day and hiding beneath cover at night. This cool and moist montane environment appears to be essential for sustaining the species. From a conservation standpoint, *Scincella tenuistriata* sp. nov. is currently known only from its type locality in Qixingguan District, Guizhou Province. The restricted distribution and limited number of specimens highlight its potential vulnerability to habitat disturbance. However, given the absence of data on population size, trends, and wider distribution, we recommend that the species be preliminarily assessed as Data Deficient (DD) under the IUCN Red List criteria. Future surveys in adjacent montane areas are necessary to clarify its conservation status and to determine whether it may warrant listing in a threatened category.

## Supplementary Material

XML Treatment for Scincella tenuistriata

## ﻿Additional information

### Use of AI

No use of AI was reported.

### Author ORCIDs

Yuhao Xu https://orcid.org/0000-0001-6094-6680

Tan Van Nguyen https://orcid.org/0000-0001-5413-968X

Andrey M. Bragin https://orcid.org/0000-0002-3621-9763

Tierui Zhang https://orcid.org/0009-0000-1869-9313

Nikolay A. Poyarkov https://orcid.org/0000-0002-7576-2283

Lifang Peng https://orcid.org/0000-0001-9325-7048

### Data availability

All of the data that support the findings of this study are available in the main text.
